# The Influence of Glow and Afterglow Cold Plasma Treatment on Biochemistry, Morphology, and Physiology of Wheat Seeds

**DOI:** 10.3390/ijms23137369

**Published:** 2022-07-01

**Authors:** Pia Starič, Jure Mravlje, Miran Mozetič, Rok Zaplotnik, Barbara Šetina Batič, Ita Junkar, Katarina Vogel Mikuš

**Affiliations:** 1 Jožef Stefan Institute, Jamova cesta 39, 1000 Ljubljana, Slovenia; miran.mozetic@ijs.si (M.M.); rok.zaplotnik@ijs.si (R.Z.); ita.junkar@ijs.si (I.J.); katarina.vogelmikus@bf.uni-lj.si (K.V.M.); 2 Biotechnical Faculty, University of Ljubljana, Jamnikarjeva ulica 101, 1000 Ljubljana, Slovenia; jure.mravlje@bf.uni-lj.si; 3 Institute of Metals and Technology, Lepi pot 11, 1000 Ljubljana, Slovenia; barbara.setina@imt.si

**Keywords:** cold plasma, glow, afterglow, plants, seed, wheat, XPS, FTIR, SEM, AFM, germination, α-amylase

## Abstract

Cold plasma (CP) technology is a technique used to change chemical and morphological characteristics of the surface of various materials. It is a newly emerging technology in agriculture used for seed treatment with the potential of improving seed germination and yield of crops. Wheat seeds were treated with glow (direct) or afterglow (indirect) low-pressure radio-frequency oxygen plasma. Chemical characteristics of the seed surface were evaluated by XPS and FTIR analysis, changes in the morphology of the seed pericarp were analysed by SEM and AFM, and physiological characteristics of the seedlings were determined by germination tests, growth studies, and the evaluation of α-amylase activity. Changes in seed wettability were also studied, mainly in correlation with functionalization of the seed surface and oxidation of lipid molecules. Only prolonged direct CP treatment resulted in altered morphology of the seed pericarp and increased its roughness. The degree of functionalization is more evident in direct compared to indirect CP treatment. CP treatment slowed the germination of seedlings, decreased the activity of α-amylase in seeds after imbibition, and affected the root system of seedlings.

## 1. Introduction

Seeds, such as legumes and cereals, play an essential role in human nutrition. They are a vital source of protein, starch, oil reserves, and essential minerals. These components provide essential nutrients in the early stages of seed germination, growth, and development [1]. Increasingly unstable weather and environmental changes impact the seed germination, growth, development, and especially yield of essential cultivars, such as wheat [2,3]. To tackle these unfavourable conditions, new environmentally friendly technologies in agriculture are being developed to improve the germination and growth of seeds and, at the same time, reduce the use of pesticides, fertilisers, and other agrochemicals impacting the environment. One such new technology is the cold plasma treatment of seeds [4,5].

Cold or non-thermal plasma (CP) is ionized gas composed of electrons, positively charged ions, radicals, gas atoms, molecules, and photons, including ultraviolet (UV) or vacuum ultraviolet (VUV) radiation. CP treatment of seeds in low-pressure reactors could also be accompanied by slight fluctuations in the temperature and exposure to vacuum conditions [6,7,8]. Depending on the CP system, seeds (or other materials) could be exposed to different regimes, for example, to the glow (direct CP treatment) or afterglow (indirect CP treatment). CP components such as electrons, positively charged ions, shorter- and longer-lived radicals, and UV and VUV radiation are all part of the glow regime. The samples in this regime are exposed to chemical species that are highly reactive and aggressive. In the afterglow regime, however, only longer-lived, less aggressive chemical species are present. As a result, indirect plasma treatment in the afterglow regime is weaker, which requires longer treatment periods and induces less impairment to biological materials [9,10,11].

There has been an increase in research concerning the CP treatment of seeds for improved germination, growth, yield, and even increased resistance to abiotic stress. The research has been done on many plant species and important plant crops for human nutrition [12,13,14,15,16]. One such crop is wheat, which presents the main source of calories and proteins of non-meat origin [3]. Because of its large use and consumption, wheat was chosen as a model plant species in this research. There has been quite a few published research articles dealing with CP treatment of wheat seeds and are summarised and described in a review article written by Scholtz et al. [17]. Recently, some advances were also made in wheat seed treatment with plasma-activated water and also showed promising results for the future [18,19]. The results of CP treatment of wheat seeds are variable. For example, the CP treatment could decrease [20,21,22] or increase [23,24,25] the germination rate of seeds or cause no effects [22,26]. Some researchers also report different response mechanisms to CP treatment of different varieties of the same species. There might be no significant effect in one wheat variety on seed germination rate. However, other varieties may show a strong decrease in seed germination rate, indicating a higher sensitivity to CP treatment [20]. Such findings were also reported on other plant species, such as oilseed rape, rice, and pea seeds [21,27,28]. The effects of CP treatments on wheat seedling root and shoot growth and development were also inconclusive. Most of the researchers reported increased shoot height [23,24,25,29,30]. A more variable change after plasma treatment was seen in the number and length of wheat seedling roots, where either no or negative changes were recorded or an increase in root length was seen [17]. 

There are also missing data on how different plasma parameters influence seed surface properties, especially in terms of a more quantitative evaluation of seed surface roughness and chemistry. Most of the morphological characteristics of wheat seed surface after CP treatment were studied by SEM [31,32,33]. However, techniques such as atomic-force microscopy (AFM), not yet employed in this kind of studies, can help us to quantitatively evaluate the changes in the roughness of the surface. Chemical changes on the wheat seed surface were previously evaluated by Guo et al. [34], where the researchers reported a decrease in lipid groups and an increase in polar groups on the seed surface after plasma treatment. Similar findings were also reported on maize seeds [35] and seeds of *Arabidopsis thaliana* [36]. Studies comparing the glow (direct) and afterglow (indirect) plasma treatment are also scarce [20,37] but provide some important insights into the interaction mechanisms of CP components with the seed surface.

In the following study, we present a detailed investigation of chemical changes caused by glow and afterglow CP treatment in wheat seeds, as analysed by XPS and FTIR. Morphological characteristics were evaluated with SEM and AFM analysis. The evaluated morphological and chemical characteristics enable us to determine which plasma effects contribute to the increased water uptake and wettability of the seeds. To evaluate how these surface changes affected the physiology of seeds, germination tests, growth studies and measurement of α-amylase activity were performed.

## 2. Results

### 2.1. Water Contact Angle

**Figure 1:**
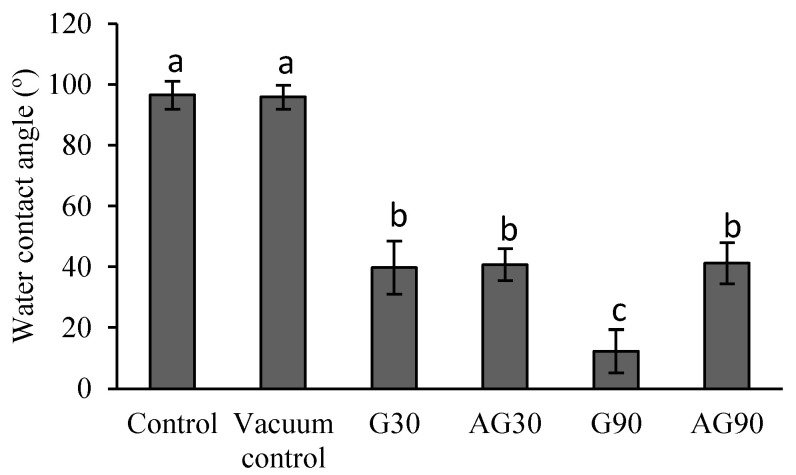
Water contact angle on the wheat seed surface of untreated (control), vacuum-treated seeds (vacuum control), and seeds treated in the CP glow regime for 30 s (G30) or 90 s (G90) and afterglow regime for 30 s (AG30) or 90 s (AG90). Displayed values are the mean ± SE of three replications. Different letters (a–c) indicate statistically significant differences among treatments according to Duncan’s test (*p* < 0.05).

Chemical and morphological changes on the seed surface after plasma treatment could be detected by measuring the changes in water contact angle (WCA). The results are presented in Figure 1. A decrease in WCA was correlated with the increased hydrophilization of the surface. A vacuum condition did not change the WCA and thus did not contribute to the changes seen in CP-treated seeds. These, on the other hand, exhibited a significant decrease in WCA compared to the controls. No significant differences were detected between glow and afterglow CP-treated seeds for 30 s (G30 and AG30). However, a significant difference was observed in glow- and afterglow-treated seeds for 90 s (G90 and AG90). Glow treatment for 90 s caused even more evident decrease in WCA than the afterglow regime. This could be explained with more intense surface etching, resulting in removal of the hydrophobic lipid layer on the seed surface and also to nanostructuring of the surface. However, all CP-treated seed surfaces exhibited a hydrophilic character, significantly contributing to seed germination and growth through better water uptake. 

### 2.2. SEM and AFM Analysis of the Seed Surface Morphology

**Figure 2:**
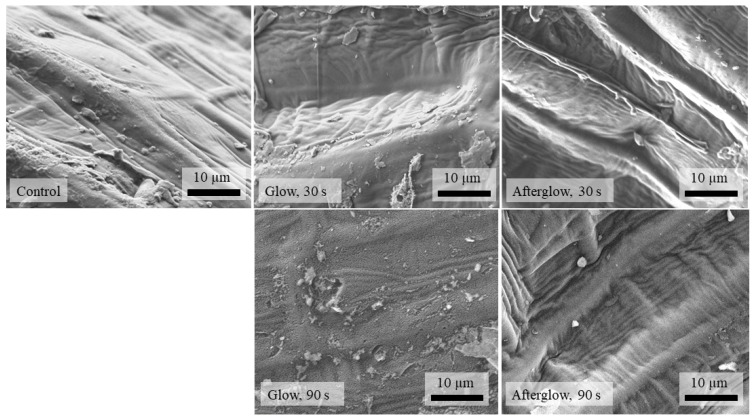
Representative SEM images of control wheat seeds and seeds treated with glow and afterglow CP for 30 s and 90 s.

The effect of CP treatment of seeds can be seen in the changed morphology of the seed surface. Scanning electron microscopy (SEM) provides a view of the seed surface morphology under high magnification. In our study, the control (untreated) and CP-treated seed surface in the glow or afterglow regime for 30 s and 90 s treatment (Figure 2) was analysed. Results showed that the control surface was inhomogeneous with different microsized lines present on the surface. A similar morphology was observed also for treatment in the glow regime for 30 s and both afterglow regimes (30 s and 90 s). It should be emphasised that no changes in surface morphology after vacuum exposure were observed; thus, SEM and AFM images were not added for the vacuum control samples. Only the seed surfaces of samples treated in glow regime for 90 s exhibited a change in surface morphology towards a more nanostructured surface. Thus, the etching effect on the seed surface after plasma treatment was in our case observed only after longer direct (glow) plasma treatment.

**Figure 3:**
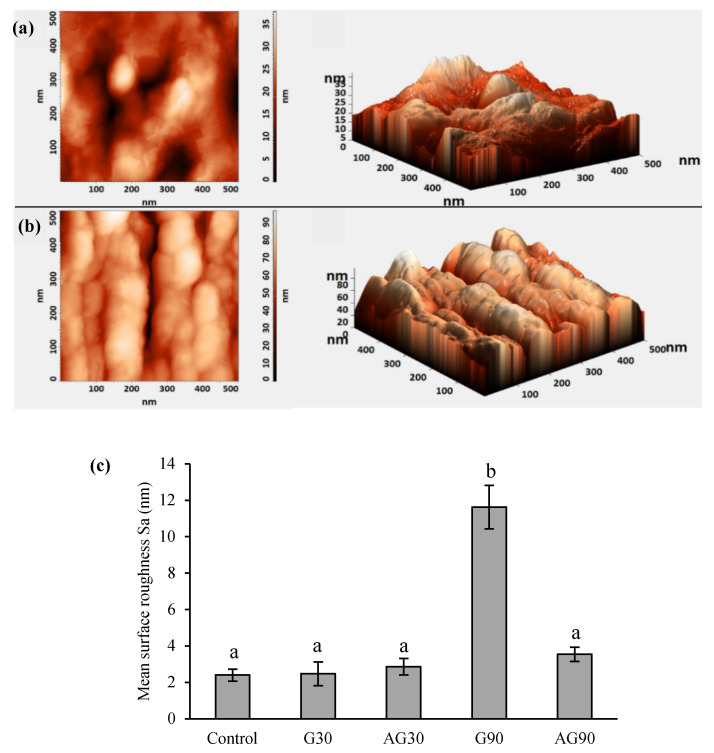
AFM height and 3D image of seed coat surface of untreated seeds (**a**) and CP-treated seeds in the glow regime for 90 s (G90) (**b**). Mean surface roughness (Sa) of seeds treated in glow regime for 30 (G30) or 90 s (G90), and afterglow regime for 30 (AG30) or 90 s (AG90) (**c**). Displayed values are the mean ± SE of three replications. Different letters (a, b) indicate statistically significant differences among treatments according to Duncan’s test (*p* < 0.05).

Examination of the seed surface with atomic force microscopy (AFM) exhibited a notable increase in roughness of the seed coat of seeds treated in G90 (Figure 3c). At other treatment conditions, no significant changes in mean surface roughness were observed. In Figure 3a (control) and Figure 3b (G90), the AFM height and 3D image of seed surface morphology is presented, where an increased surface roughness with higher peaks (up to 80 nm) is seen on the seed coat of G90. On the other hand, the surface of the control seed was less structured and had lower crests (up to 35 nm in height). Other plasma treatments did not exhibit any changes in surface roughness compared to the control (Appendix A). The evaluated surface roughness (Sa) was about 2–4 nm for all seeds except for the G90 seed surface, where the average roughness was about 12 nm, as seen in Figure 3c.

### 2.3. Chemical Analysis of Seed Surface by XPS Spectroscopy

Modifications in seed surface chemistry were analysed by X-ray photoelectron spectroscopy (XPS), providing information on the atomic composition of elements on the top surface about 5 nm in depth. The surface of the CP-treated seeds contained a lower amount of carbon (C) and a higher amount of oxygen (O) compared to control samples (untreated seeds) (Table 1). The vacuum control sample did not differ from the control sample; thus, it was not added to Table 1. The G90 treatment significantly increased oxygen content in comparison to G30, since functionalization of the seed surface with oxygen reactive chemical species was higher. The large oxygen concentration, as determined by XPS, was partly due to the formation of oxides, while the pronounced increase in potassium (K), sulphur (S), magnesium (Mg), and calcium (Ca) was due to selective etching, which removed the top lipid layer and uncovered the microelements below. In G30 treatment, a much higher concentration of oxygen on the seed surface was observed than in AG30. This was expected, as the afterglow regime is characterised by a lower density of reactive oxygen species and absence of ions that bombard the surface and increase the surface roughness, increasing the area for functionalization. Even more pronounced changes in surface chemistry were observed in G90 and AG90, causing much more pronounced surfaces. The increase in concentration of potassium (K), sulphur (S), magnesium (Mg), calcium (Ca), and silicon (Si) was due to plasma treatment. K, Ca, and Si were detected on CP-treated seed surfaces but not on untreated seeds. Evidently, direct and longer CP treatments cause more pronounced chemical changes on seed surfaces. 

### 2.4. Fourier Transform Infrared Spectroscopy Analysis of Chemical Changes on the Seed Surface

**Figure 4:**
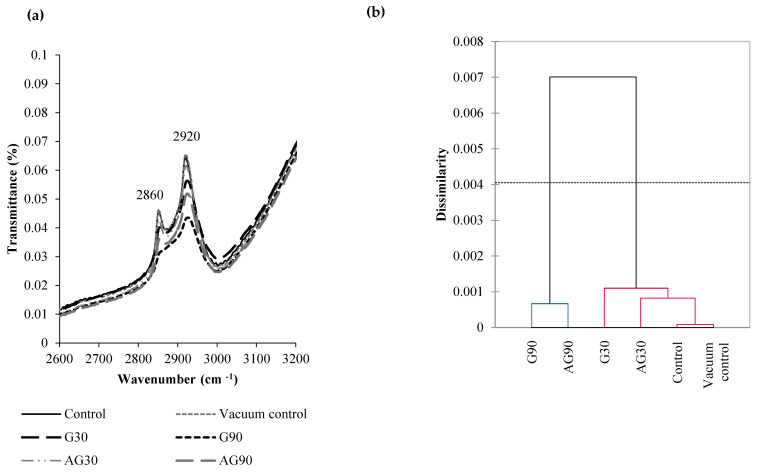
(**a**) FTIR spectroscopy spectrum of untreated seeds (Control), seeds exposed to vacuum conditions (Vacuum control), and seeds treated in the CP glow regime for 30 s (G30) or 90 s (G90), and the afterglow regime for 30 s (AG30) or 90 s (AG90). Dendrogram of FTIR absorbance in the 2600–3200 cm^−1^ wavelength area (**b**).

The seed coat of wheat is covered by a thin layer of lipids. During plasma treatment, the seed surface was exposed to functionalization with oxygen species, as confirmed by XPS analysis. WCA analysis showed that the surface of seeds became more hydrophilic after CP treatment, and XPS analysis of the seed surface confirmed the oxidation of the molecules, since the oxygen content increased, and carbon content decreased. To confirm the oxidation of lipid molecules, Fourier transfer infrared (FTIR) spectroscopy was performed on seed coat material. The 2820–2980 cm^−1^ range is associated with the C–H stretching vibrations of alkenes or lipid molecules [14]. Control and vacuum control seeds retained the infrared spectral profile in the lipid area, so the vacuum conditions did not affect the chemical composition of the lipids on the seed surface (Figure 4a). Treatment of the seeds in G30 caused a slightly lower transmittance in the 2860 cm^−1^ and 2920 cm^−1^ peaks, as the oxidation of lipids took place. AG30 treatment, on the other hand, showed a smaller decrease in these peaks, as the functionalization with oxygen was much weaker in the afterglow regime. The highest decrease in the lipid peaks was observed in G90 treatment. Again, the AG90 decreased the lipid peaks, but not as much as G90 compared to the control. Both G90 and AG90 CP regimes lowered the content of lipid groups compared to the shorter treatment time (30 s). 

The similarity among different treatments is represented in a dendrogram in Figure 4b. The dendrogram distribution for the FTIR absorbance values in the 2600–3200 cm^−1^ wavelength area (lipids) showed that a clade with control and vacuum samples was the most similar to samples treated in the afterglow regime for 30 s, followed by 30 s treatment in the glow regime. The 90 s treatment in the CP (glow or afterglow regime) gave substantially different values from the other samples. Thus, the changes in the oxidation of lipids on the seed surface were significantly different in longer treated samples (90 s), when compared to the control samples.

### 2.5. Plasma Treatment Increases Water Uptake in Wheat Seeds

**Figure 5:**
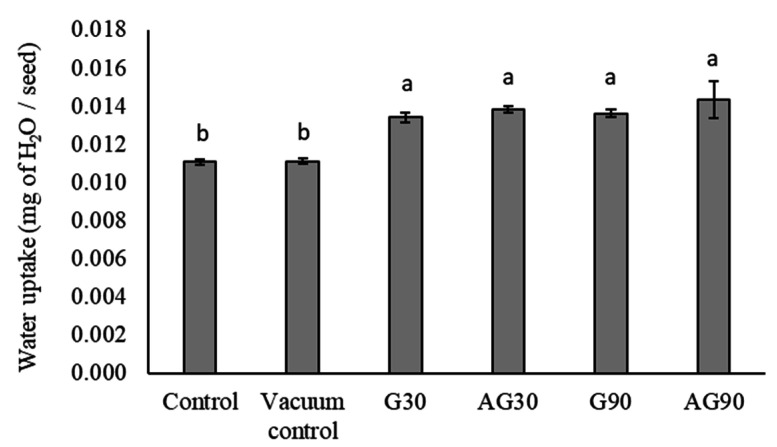
Water uptake of the Control (untreated), seeds exposed to vacuum conditions (Vacuum control) and CP-treated seeds in the glow regime for 30 (G30) or 90 s (G90), and the afterglow regime for 30 (AG30) or 90 s (AG90). Displayed values are the mean ± SE of three replications. Different letters (a, b) indicate statistically significant differences among treatments according to Duncan’s test (*p <* 0.05).

Chemical changes after CP treatment made the seed surface more hydrophilic, affecting the water uptake of seeds upon imbibition. We measured the water uptake of control and CP-treated seeds (Figure 5). The vacuum conditions alone did not affect the water uptake of the seeds as there were no significant changes compared to the control. Water uptake of treated seeds, however, increased, but no significant changes were noticed between the different CP treatments.

### 2.6. Moisture Content in Seeds after CP Treatment

**Figure 6:**
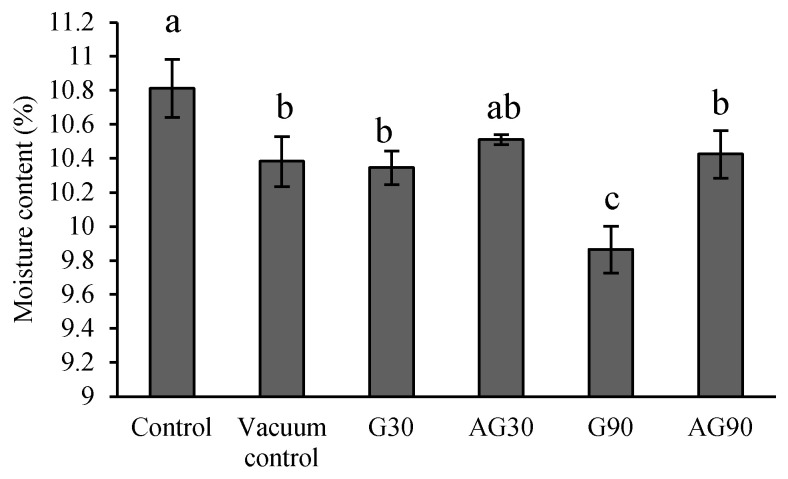
Moisture content in untreated (Control) seeds, seeds exposed to vacuum conditions (Vacuum control), and CP-treated seeds in the glow plasma regime for 30 (G30) or 90 s (G90), and in the afterglow regime for 30 (AG30) or 90 s (AG90). Displayed values are the mean ± SE of three replications. Different letters (a–c) indicate statistically significant differences among treatments according to Duncan’s test (*p <* 0.05).

Sufficient but not excess moisture content (MC) in seeds is essential for seed viability and overall fitness of the later development and growth of the seedling. Seeds in our plasma system were exposed to vacuum and plasma species, which could have potentially affected the MC. The experiments showed that vacuum conditions without the CP treatment caused a significant decrease in the MC of seeds (Figure 6). A similar decrease was found in seeds treated in G30 and AG30 and AG90. A still lower MC, however, was found in seeds treated with G90. The greater loss of MC in longer treated seeds (G90) was most likely a synergistic effect of both vacuum conditions and CP treatment, probably due to the increase of seed temperature to ~80 °C during the treatment. The details are presented in Section 4.

### 2.7. Longer Direct CP Treatment Affects Seed Germination and Speed

**Figure 7:**
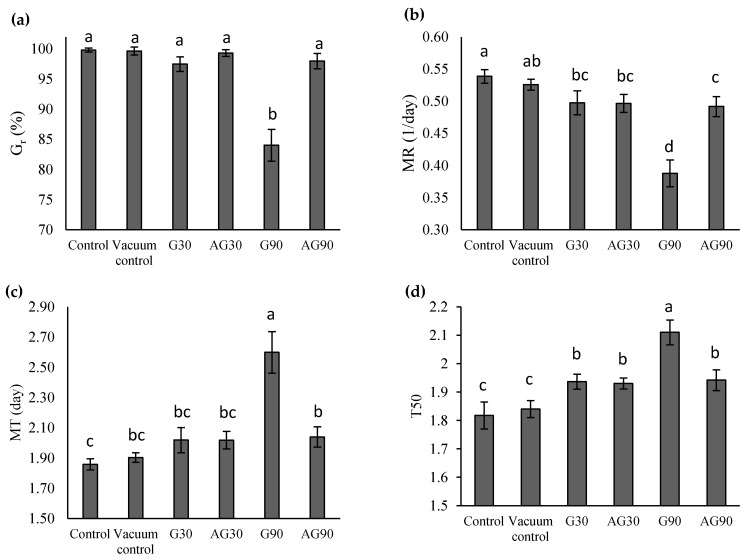
Final germination rate—G_r_ (**a**), mean germination rate—MR (**b**), mean germination time—MT (**c**), and median germination time—T50 (**d**) of untreated (Control) seeds, seeds exposed to vacuum (Vacuum control), and CP-treated wheat seeds in the glow regime for 30 (G30) or 90 s (G90), and the afterglow regime for 30 (AG30) or 90 s (AG90). Displayed values are the mean ± SE of three replications. Different letters (a–d) indicate statistically significant differences among treatments according to Duncan’s test (*p* < 0.05).

CP treatment of seeds can have harmful, neutral, or positive effects on seed germination. To assess how our discharge parameters of plasma treatment affected the seeds germination, we determined final germination rate (G_r_), mean germination rate (MR), mean germination time (MT), and median germination time (T50). CP treatment did not significantly affect the final germination rate, except for the seeds treated in the G90 regime, where the germination rate decreased significantly (Figure 7a). 

From the mean germination rate (MR) calculations (also called index of germination speed), the MR was lower in CP-treated seeds (Figure 7b). This showed that the treatment slowed the germination of seeds, especially the seeds treated in the G90 regime. Seeds treated in the AG90 regime germinated faster.

Mean germination time calculations showed that only the G90-treated seeds exhibited a higher MT than the controls (Figure 7c). The G90 seeds showed a significantly higher T50 than the controls and other CP treatments (Figure 7d). For AG90 seeds, T50 was lower in comparison. All CP treatments exhibited significantly higher T50 than the control. This correlates with the lower MR of plasma-treated seeds.

### 2.8. α-Amylase Activity in Seeds 

**Figure 8:**
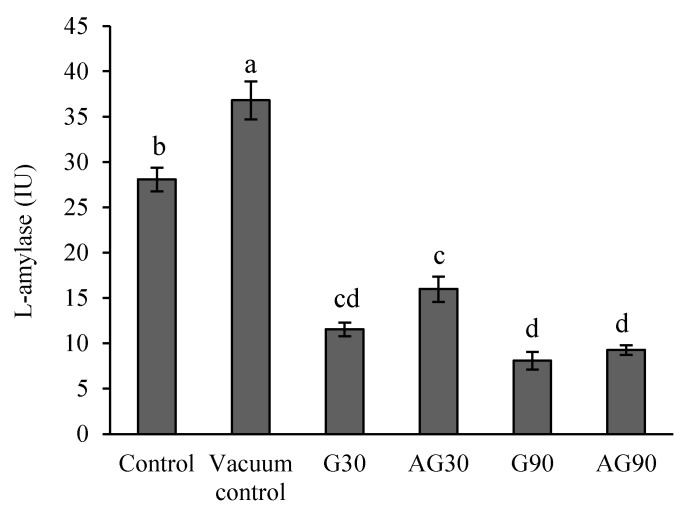
The activity of α-amylase enzyme in untreated (Control) seeds, seeds exposed to vacuum (Vacuum control), and CP-treated seeds in the glow regime for 30 s (G30) or 90 s (G90), and in the afterglow regime for 30 s (AG30) or 90 s (AG90). Displayed values are the mean ± SE of three replications. Different letters (a–d) indicate statistically significant differences among treatments according to Duncan’s test (*p* < 0.05).

A crucial process involved in seed germination after the imbibition of the seeds is starch decomposition, providing fuel to the seed embryo for its development and growth [15]. One of the main enzymes accountable for starch degradation is α-amylase, activated during the seed imbibition. As shown in Figure 8, seeds exposed to vacuum conditions showed significantly higher α-amylase activity than the controls. All plasma treatments, however, caused a decrease in α-amylase activity, but without a difference between glow and afterglow treatment for the same treatment time (30 s or 90 s). Thus, a common component of direct and indirect plasma treatment apparently affects the activity of α-amylase in wheat seeds.

### 2.9. Root Length and Number of Roots of Seedlings

**Figure 9:**
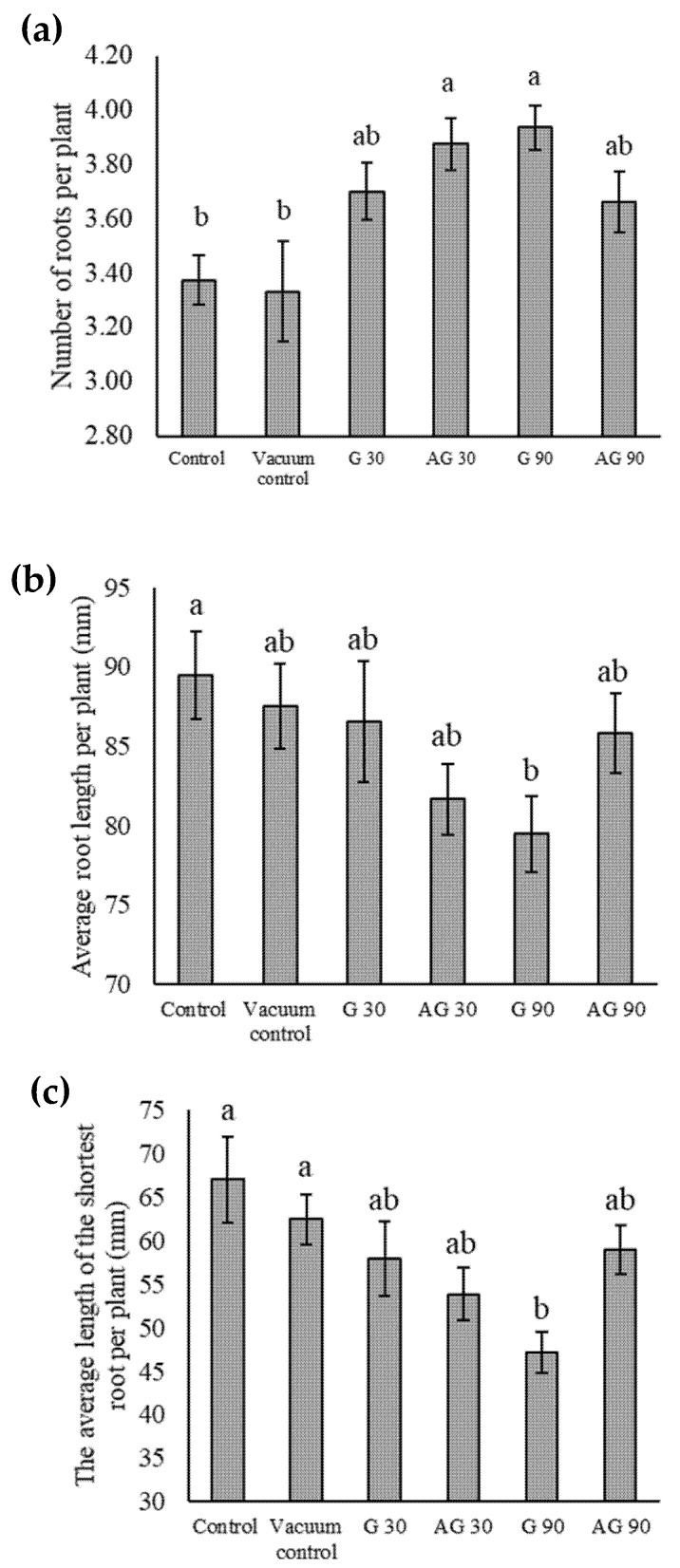
The average number of roots per seedling (**a**), the average root length per seedling (**b**), and the average length of the shortest root per seedling (**c**) in untreated (Control) seeds, seeds exposed to vacuum (Vacuum control), and CP-treated seeds in the glow regime for 30 (G30) or 90 s (G90), and in the afterglow regime for 30 (AG30) or 90 s (AG90). Displayed values are the mean ± SE of three replications. Different letters (a, b) indicate statistically significant differences among treatments according to Duncan’s test (*p <* 0.05).

In addition to changes in seed germination parameters, CP could also affect the development of seedlings. Wheat has a seminal root system in young seedlings [16]. We noticed a statistically significant effect of CP on the decrease in length of the shortest root per plant (Figure 9a) and average root length (Figure 9b) of the seedlings raised from seeds treated in the G90 regime. There was also an increase in the number of roots per seedling (Figure 9c) in AG30 and G90. The total sum of root length per seedling, as well as the length of the longest seedling root from treated seeds, did not show any statistically significant changes compared to the control (Appendix B). We also did not detect any statistically significant differences in seedling shoot length or weight between CP treated and untreated seeds.

## 3. Discussion

CP treatment of seeds has been known to affect the hydrophobic properties of the seed coat of a number of plant species, including wheat. It reduces the seed coat water contact angle (WCA), indicating a more hydrophilic surface after the treatment [17,18,19]. With longer plasma treatments, the WCA of the seed coat decreases over time [19], as confirmed also by our results. However, research on wheat seed treatment in the afterglow regime is lacking. We showed that shorter CP treatments in the afterglow regime affected the WCA of the seed surface similarly as the glow CP regime. However, in the longer 90 s treatments, a difference was measured between the WCA on seeds treated in the afterglow and glow regimes, with the weaker nature of the former more clearly exposed. The WCA of the AG90 treated seeds was thus similar to the WCA of both G30 and AG30 seeds, indicating that hydrophilic changes by CP achieved “saturation” already at 30 s exposure.

The changes in the WCA of the seed coat after CP treatment could be ascribed to either morphological changes or chemical functionalization of the seed surface by CP component changes or a synergistic effect of both. Several authors found morphological changes of the seed surface after CP treatment by SEM analysis [20,21,22,23]. No research, however, has explored the changes in nanostructuring and increased roughness of the seed pericarp. Our AFM results indicated that only G90 treatment caused an increased roughness of the seed pericarp, which correlated with our results from the SEM analysis. The changed morphology results from the etching in plasma [10]. Thus, the WCA decrease of plasma-treated seeds in G30, AG30, and AG90 was probably only due to the functionalization of the seed surface and changed chemistry and not due to changes in seed morphology. The even lower decrease in WCA in seeds treated in G90 shows a synergistic effect of morphological and chemical changes on the seed surface.

Analysis of chemical changes after CP treatment by XPS and FTIR showed oxidation of lipids. This contributed to the lowered WCA of the CP-treated samples. The XPS spectrum showed also an increased potassium content after all CP treatments, especially after longer CP exposures. The results correlate with similar observations on quinoa and nasturtium seeds [24,25]. However, our results also showed the presence of Ca and Si after all CP treatments, which was not seen in the control samples. Ca and Si are typical elements of the wheat pericarp [26,27]. The XPS analysis of G90 and AG90 treatments also detected S, Mg, and P, not detected in control samples and samples treated in plasma for 30 s. The CP treatment possibly removed the outermost waxy layers and exposed elements from the deeper layers of pericarp.

The hydrophilic change in the seed coat contribute to the higher water uptake of seeds; the increased water uptake of plasma-treated seeds has been reported previously [28,29]. Vacuum conditions during plasma treatment of seeds could potentially influence the moisture content of seeds [30]. However, the results of experiments conducted by Los et al. [13] on wheat seeds showed no significant changes in moisture content of plasma-treated seeds. Our results, however, indicated that vacuum itself negatively influenced the seed moisture. Plasma treatment of seeds in G90 additionally decreased the moisture content in seeds. Thus, vacuum conditions and a prolonged exposure to plasma cause a decrease in moisture content.

Germination studies showed no changes in germination rate except for the G90 regime, where the final germination rate was significantly lowered compared to the control, with a lower speed of germination. Apparently, the dose of plasma components in this treatment surpassed the optimum seed threshold. There was no significant improvement of seed germination treated under other CP conditions, as the germination rate of the untreated seeds was already high (~99%). Other authors [20,21,31,32], however, report an improvement of wheat germination rate after CP treatment. However, in the case of Los et al. [19], the germination rate of control seeds was rather low (~65%). In addition, the authors used a different kind of plasma system and different wheat varieties, which makes the comparison of the results inconclusive. Other authors reported no significant effects of plasma treatment on seed germination [33,34,35].

α-Amylase activity measured in the seeds treated with plasma was lower than in untreated seeds. The α-amylase enzyme is responsible for the degradation of starchy food reserves in endosperm during germination. The enzyme is synthesised de novo in the aleurone layer of seeds upon imbibition through hormonal regulation of gibberellins [36,37]. Lower detected activity of this enzyme can indicate a delay in enzyme activation, which correlates with a slower germination of the CP-treated seeds shown by mean germination time (MT) and median germination time (T50). Thus, the signalling pathway to activate α-amylase was affected directly or indirectly by CP treatment. It could either affect the gene expression of the α-amylase enzyme or influence the (in)activation of the enzyme itself. An increase in α-amylase activity in CP-treated rice seeds has been reported [29]. However, the CP treatment caused a faster germination of rice seeds, possibly due to the increase in α-amylase activity, as the enzyme and germination process are closely related. The comparison of the results is inconclusive, as the plasma reactors and plants used in these experiments are different. Several studies also showed that seed responses to plasma treatment might be plant- and variety-dependent in many crop species [12,38].

Interestingly enough, the exposure to vacuum conditions enhanced the enzyme activity for reasons yet to be investigated. Furthermore, there was a decrease in the enzyme activity upon treatment in both glowing plasma and afterglow. The effect cannot be explained by heating of the seeds, since the seed temperature remained unaffected by treatment in the afterglow region. The difference in our afterglow reactor and the reactor used in [29] is in the O-atom density. While our reactor enabled an O-atom density as large as 1 × 10^21^ m^−3^, the atom density in the reactor of [29] should be much lower because of a DC discharge at a very low power of a few W only. Unfortunately, the authors [29] did not report the O-atom density, but from general principles [39] it should be orders of magnitude lower than in our reactor. The results presented in Figure 8 may indicate that the exposure of the seeds to large fluences of O-atoms suppresses the α-amylase activity.

Even though there were no evident changes in seed germination, changes in the other stages of plant development can appear. CP-treated seeds exhibited a higher number of roots, indicating that the plant metabolism was affected. The increase in the number of roots could indicate the presence of stressor(s) as exposure to certain stressors, such as hydrogen peroxide (H_2_O_2_) [40]. It has been proposed that CP acts as a stressor. The appropriate level of such treatment can thus result in a positive stress response in plant metabolism, enhancing seedling and plant growth, stress tolerance, and increased yield [38]. However, in the case of CP-treated wheat seeds, the reports only gave the increase in root length of seedlings [33,41], in contrast with our results, where the length of the roots decreased, especially after CP treatment in the G90 regime. Rahman et al. [21] also reported a decrease in root length in plasma-treated wheat seeds.

**Figure 10:**
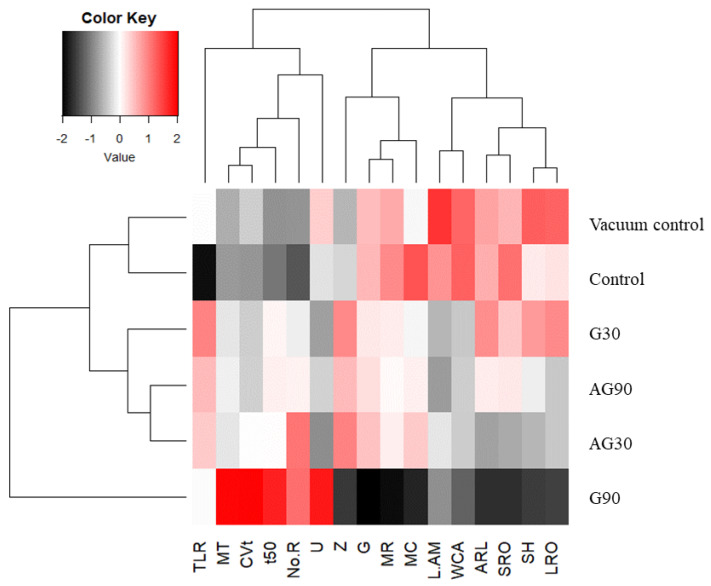
A two-way clustering analysis of the measured wheat parameters: Total root length per plant (TLR), mean germination time (MT), coefficient of variation of the germination time (CVt), median germination time (t50), number of roots per plant (No.R), the uncertainty of the germination process (U), synchrony of germination (Z), germination rate (G), mean germination rate (MR), moisture content (MC), α-amylase activity (L.AM), water contact angle (WCA), average root length per plant (ARL), the average length of the shortest root (SRO), shoot height (SH), and average length of the longest root (LRO). Samples seen on the right side of the heatchart are as follows: untreated seeds exposed to vacuum conditions (Vacuum control), untreated seeds (Control), and CP-treated in glow regime for 30 (G30) or 90 s (G90), and in afterglow regime for 30 (AG30) or 90 s (AG90).

To summarize the findings of this study, we performed a two-way clustering analysis and presented it in a heatmap (Figure 10). The treatment of seeds in the G90 regime produced the most distinct heatmap pattern compared to the control and vacuum control.

## 4. Materials and Methods

### 4.1. Seed Material and Plasma Treatment

Seed material of winter wheat (*Triticum aestivum* L. cv. “Ingenio”) was supplied by the Agricultural Institute of Slovenia and stored under room conditions. Seeds were not pre-treated with any chemicals.

**Figure 11:**
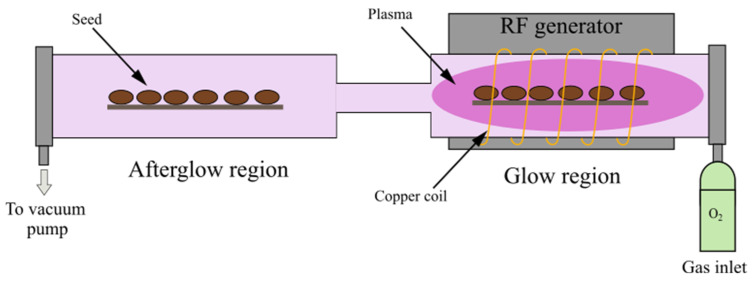
A schematic representation of the plasma reactor used in the experiment.

We chose a small-scale CP system using low pressure inductively coupled (ICP) radio-frequency (RF) oxygen plasma. Plasma was powered with an RF generator operating at the standard industrial frequency of 13.56 MHz and adjustable output power up to about 1000 W. The plasma reactor is schematically presented in Figure 11. Plasma formed inside the glass tube of inner diameter 36 mm within six turns of copper coil, connected to the RF generator via a matching network. After a narrow tube section of 80 mm length and 7 mm diameter, the afterglow regime of plasma was exploited. To maintain the desired low pressure in the system, the reactor was continuously pumped by a vacuum pump connected to the plasma reactor as illustrated in Figure 11. Oxygen of commercial purity 99.999% was introduced on the other side of the experimental system during continuous pumping. The narrow tube supressed the effective pumping speed of the two-stage rotary vacuum pump of nominal pumping speed 80 m^3^/h so that a pressure gradient was established. The plasma was formed under a working pressure of 50 Pa at a forward power of 200 W. The reflected power was roughly 100 W, so the power absorbed by plasma electrons was below 100 W. The pressure in the afterglow part of the experimental system was 15 Pa. Seeds were treated for 30 and 90 s in the direct (glow) regime and indirect (afterglow) regime.

Plasma was characterized by optical spectroscopy and by electrical and catalytic probes. The temperature of seeds on a perforated aluminium holder was estimated with an infrared pyrometer operating at wavelengths of 8 to 14 µm. The seeds placed in the afterglow of the plasma system were not warmed for more than a few °C even after the treatment for 90 s. The samples mounted in the glowing plasma, however, were heated significantly. The samples treated for 30 s and 90 s were heated for about 40 °C and about 80 °C, respectively. Therefore, any thermal effect on the biological properties can be neglected for seeds placed in the afterglow segment of the experimental chamber but should be considered when seeds were treated in glowing plasma. 

**Figure 12:**
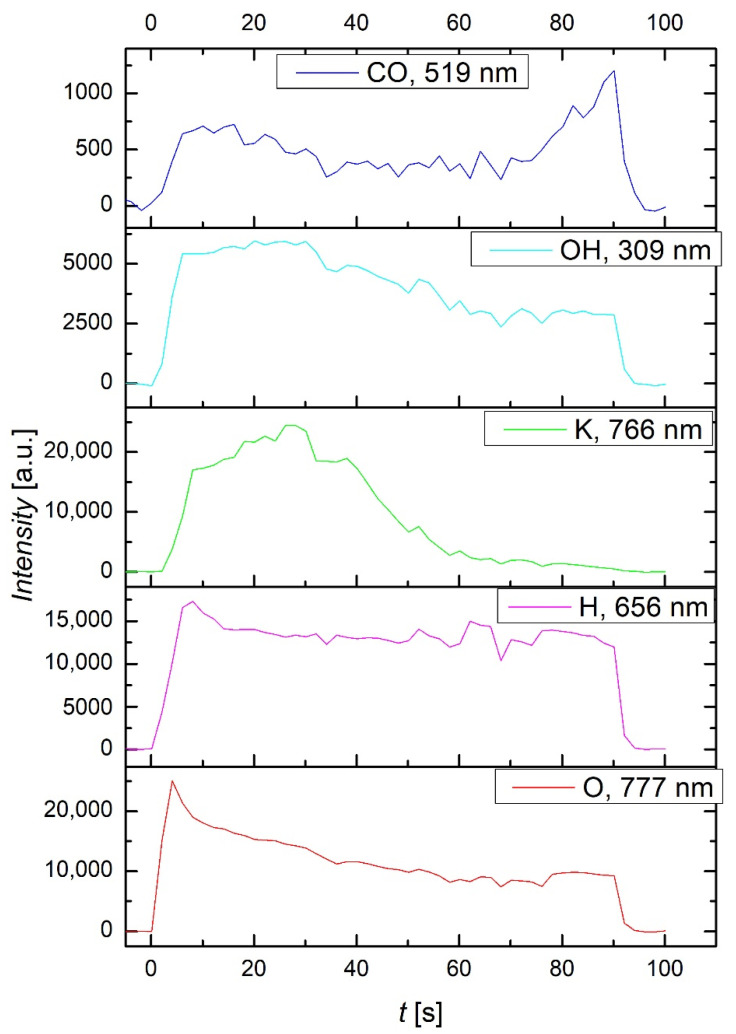
The intensity of some optical features versus plasma treatment time.

The evolution of most relevant spectral features as deduced from optical spectra is shown in Figure 12, with an integration time of 1 s. As the plasma was ignited, the main O-atom line at about 777 nm became predominant, but its intensity slowly decreased with increasing treatment time. The observation was expected, since oxygen was inertially introduced into the system. The H-atom line at 656 nm arose from partial dissociation of water molecules upon plasma conditions. The same applied for the OH band at the band head of about 309 nm. The water vapour always existed in the vacuum system, but the large intensity indicated a rather large partial pressure, explained by the desorption from the seeds. More interesting is the significant radiation arising from potassium. The K-line at 766 nm was very intensive, about the same as the O-atom line, and appeared with a short delay of a few seconds after turning on the discharge. The seed coat contained some potassium, and the quick appearance indicated etching of the organic material even at low temperature. The etching was explained by the interaction of reactive plasma species, O-atoms in the ground and metastable state, as well as positively charged ions with the seed coat. This resulted in the formation of low-weight molecular fragments, which were desorbed from the seed surface in vacuum conditions, and the fragments were further dissociated to atoms. The large intensity of the K line at 766 nm did not prove a large potassium concentration in the gas phase, since the optical emission technique is a qualitative technique. Namely, the excitation cross-section for K was much larger than for O-atoms, since the radiative transition to the ground state was resonant. On the other hand, the intensity of the CO line at 519 nm was much weaker, as revealed in Figure 12. CO was formed upon oxidation of the organic matter with reactive oxygen species, but it had numerous molecular bands in the optical spectra so the intensity of a particular line was weak. In any case, both K and CO lines indicated significant etching, which did not depend much on the seed temperature. The temperature increased monotonously with treatment time, while the intensity of these spectral features even decreased slowly with time (i.e., increasing temperature).

The density of reactive species in the glowing plasma was measured with a double electric probe and a catalytic probe. A home-made electrical probe with an electrode diameter of 2 mm was used to estimate the density of positively charged oxygen ions and the electron temperature. The I(U) characteristic of the double electrical probe was only acquired in about 60 s, so the probe did not allow for time-resolved measurements. It gave a density of about 7 × 10^15^ m^−3^. The density of neutral O-atoms 6 × 10^21^ m^−3^ was measured with a calibrated catalytic probe (Plasmadis Ltd.). Such a large density is a consequence of extensive dissociation of oxygen molecules by plasma electrons and a negligible recombination of atoms to parent molecules. The density was measured during the treatment of seeds and remained constant over the entire treatment period. The consumption of O-atoms due to chemical interaction with the organic matter was therefore marginal. 

The density of O-atoms was also measured in the afterglow region of the experimental setup shown in Figure 11. The density of 1 × 10^21^ m^−3^, i.e., six times lower than in the glowing plasma, did not depend on the treatment time. The main reason for the lower density being about three times lower pressure in the afterglow was because of the narrow glass tube between the glow and afterglow parts of the experimental setup. The rest was due to the heterogeneous surface recombination of the O-atoms on the surface of the narrow tube.

### 4.2. Water Contact Angle

The water contact angle of the seed surface was measured two hours after plasma treatment. We chose ten random seeds from each plasma treatment and both controls. The water contact angle was measured with a Drop Shape Analyzer DSA 100E (KRÜSS GmbH, Hamburg, Germany) with 1 µL MiliQ water (Stakpure, Niederahr, Germany) droplets. 

### 4.3. Water Uptake

For studies of water absorption of plasma-treated and non-treated wheat seeds, 50 seeds were weighed and placed in a petri dish (fi = 60 mm) with two layers of filter paper, soaked with 4 mL of dH_2_O. Before each measurement, the seeds were blotted with a paper towel to remove excess water. Weighing of the seeds followed 0.5, 1, 3, 6, and 24 h after imbibition. For each CP treatment and the controls, four replicates with a total number of 200 seeds per treatment (4 × 50 seeds) were prepared.

### 4.4. Moisture Content

Moisture content (MC) of the seeds was measured. For each plasma treatment and both controls, 40 seeds in two replicates were placed in a small glass petri dish (φ = 30 mm) and dried in an oven at 105 °C for 24 h. Moisture content was calculated as suggested by Los et al. [13], where W_f_ represents the final mass of 40 seeds after drying, and W_i_ represents the initial mass of the sample.

MC = ((W_i_ − W_f_) × 100%)/W_i_ (1)

### 4.5. Germination

For germination studies, 50 seeds were put in a petri dish (φ = 60 mm) with two layers of filter paper soaked with 4 mL of dH_2_O. Seeds were incubated in the dark, in controlled conditions of 12 h day/night cycle, with 19 °C in the night regime and 23 °C in the day regime. Germinated seeds were counted on days 1, 2, 3, and 7 from imbibition. Each plasma treatment and both controls involved four replicates, in total 200 seeds per treatment. With the following formula, germination rate or germination percentage (G) was calculated:

G = (n_g_ × 100%)/n_t_ (2)

where n_t_ represents the total number of seeds in a petri dish, and n_g_ represents the number of germinated seeds.

Other germination parameters, such as mean germination time (MT), mean germination rate (MR), median germination time (t50), uncertainty of the germination process (U), and synchrony of germination (Z), were calculated according to Ranal et al. [42].

### 4.6. Growth Studies

For growth studies, we used 2-day-old seedlings. For each plasma treatment and both controls, 20 random seedlings were chosen. They were put in previously prepared “Phytotest kit” (EBPI, Mississauga, ON, Canada) germination chambers, with 10 seedlings per chamber. The bottom part of the chamber was composed of a layer of filter paper, a sponge, and another layer of filter paper. The chamber was wetted with distilled water, and seeds were put on the top layer of the filter paper. The closed chamber in the vertical position was incubated for 7 days in controlled conditions, with a day/night cycle of 12 h and a temperature of 20 °C/24 °C, respectively.

After the incubation period, the seedlings were placed against a black background and photographed with graph paper for scale. The lengths of roots and shoots were evaluated with the help of ImageJ software. We measured the length of the shortest root, the length of the longest root, the average root length, and total root length per plant. We also measured the shoot length and counted the number of roots per plant.

### 4.7. α-Amylase Assay

Plasma-treated and untreated seeds were imbibed in distilled water (dH_2_O) for 24 h in controlled conditions. After imbibition, seeds were blotted with a paper towel and frozen in liquid nitrogen before lyophilization of the samples for 2 days. Dry material was then crushed with a pestle and mortar to a homogenous powder mixture. 

The samples for α-amylase assay were prepared according to the user’s manual for the CERALPHA kit (Megazyme, Wicklow, Ireland). For enzyme extraction, 0.75 g of powdered sample was weighted into the glass eprouvette of 10 mL capacity. A volume of 5 mL of extraction buffer solution (pH 5.4) was added and stirred with vortexing. The samples were then incubated in a water bath at 40 °C for 20 min and then centrifuged at 1000× *g* for 10 min.

For each sample, 0.1 mL of amylase HR reagent solution was pipetted into 2 mL microcentrifuges and pre-incubated in a water bath at 40 °C for 5 min. After incubation, 0.1 mL of extracted enzyme from the sample was added and incubated in a water bath at 40 °C for 10 min. At the end of the incubation period, 1.5 mL of stopping reagent was added and mixed by vortexing. The samples were centrifuged for 5 min at 1000× *g*. The absorbances of the samples and the reaction blank were read at 400 nm against distilled water. First, we calculated α-amylase activity in CU/g of milled samples by the following formula:

(3) $$\alpha \;Amylase\;content=\frac{A400}{incubation\;time}*\frac{total\;volume\;in\;cell}{volume\;of\;extract}*0.05525*\frac{extraction\;volume}{sample\;weight}*dilution$$

After calculating α-amylase activity in CU/g of milled sample, we multiplied it by the constant 4.1 to obtain values in international units of starch substrate (IU).

### 4.8. SEM Sample Preparation

Samples for scanning electron microscopy were fixed on stubs with carbon tape. To ensure conductivity, the samples were sputter-coated using a GATAN PECS 652 system (Pleasanton, CA, USA) with an Au/Pd target. The coating thickness was approximately 5 nm. The samples were then imaged using a ZEISS CrossBeam 550 (Dresden, Germany) scanning electron microscope using 5 kV acceleration voltage and 100 pA beam current. 

### 4.9. XPS Analysis of Seed Surface

After plasma treatment, the seed was cut at the top quarter, and the seed coat was analysed by X-ray photoelectron spectroscopy (XPS). The analysis was performed with a PHI-TFA XPS spectrometer Physical Electronics Inc. (Chanhassen, MN, USA) equipped with an Al-Kα mono-chromatic source. The analysed area was about 0.4 mm in diameter. High-energy resolution spectra were acquired with an energy analyser operating at the resolution of 0.6 eV and pass energy of 29 eV. Quantification of surface composition was performed using XPS peak intensities, considering relative sensitivity factors provided by the instrument manufacturer [43].

### 4.10. FTIR Sample Preparation and Analysis

Fourier transform infrared spectroscopy (FTIR) spectra were measured on a thin section of seed pericarp with an ALPHA Routine spectrometer(Bruker, Billerica, MA, USA) at room temperature in attenuated total reflection mode in the range between 4000 cm^–1^ and 500 cm^–1^. Typically, 256 scans were recorded, averaged, and apodized with the Happ–Genzel function. The pericarp section covered the entire diamond surface. Optimum contact between the sample and the diamond was ensured by the self-levelling sapphire pressure anvil. The spectra were corrected by extended the ATR correction function and vector normalised with Opus 7.0 software (Bruker, Billerica, MA, USA).

### 4.11. Statistical Analysis

The results of the study were presented as mean ± standard error (SE) of three biological repeats. Statistical significance between the groups of samples was determined using one-way analysis of variance (ANOVA) with Duncan’s post hoc test (XLSTAT 2021). The significance level was considered at a *p*-value < 0.05. Statistically significant differences between samples were marked with different letters (a–d). The heatmaps were constructed in “R” on z-normalized data of the measured parameters. The clustering analysis was based on Euclidian distances. 

## 5. Conclusions

Our research shows that only prolonged low-pressure oxygen plasma treatment in the glow regime affects the morphology of the wheat seed pericarp. Morphological changes may be accompanied by other factors, negatively impacting seed germination and root development. Plasma treatment in optimal conditions primarily causes functionalization of the wheat seed surface with oxygen functional groups, mainly oxidising the lipid molecules naturally present on the seed surface. This is seen as a decrease in water contact angle and increased water uptake of seeds, as the water penetrates the seed pericarp more easily. The degree of functionalization is more evident in the glow than in the afterglow regime, where surface functionalization and surface etching is less pronounced. CP treatment slowed the germination of seedlings and caused lower activity of α-amylase enzyme in seeds after imbibition, indicating CP interruption of the signalling pathway that activated α-amylase enzyme production.

## Figures and Tables

**Table 1:** Atomic share of the elements of control (untreated), vacuum control, and CP-treated seeds obtained from XPS. Seeds were treated in the glow regime for 30 (G30) or 90 s (G90) and in the afterglow regime for 30 (AG30) or 90 s (AG90). Displayed values are the means of three replications. Different letters (a–d) indicate statistically significant differences among treatments according to Duncan’s test (*p* < 0.05).

Plasma Treatment	C	O	N	K	S	Mg	Ca	Si	P
Control	86.575 a	11.850 d	1.575 b	/	/	/	/	/	/
G30	65.300 c	29.033 b	3.767 a	0.333 b	/	/	0.533 bc	1.067 a	/
AG30	75.733 b	20.633 c	1.600 ab	0.633 b	/	/	0.267 cd	1.100 a	/
G90	65.300 c	48.900 a	1.250 b	5.750 a	2.150 a	1.150 a	1.100 a	0.700 ab	0.450 a
AG90	65.050 c	29.850 b	2.500 ab	0.500 b	0.300 b	0.200 b	0.750 ab	1.100 a	/

**Figure A1:**
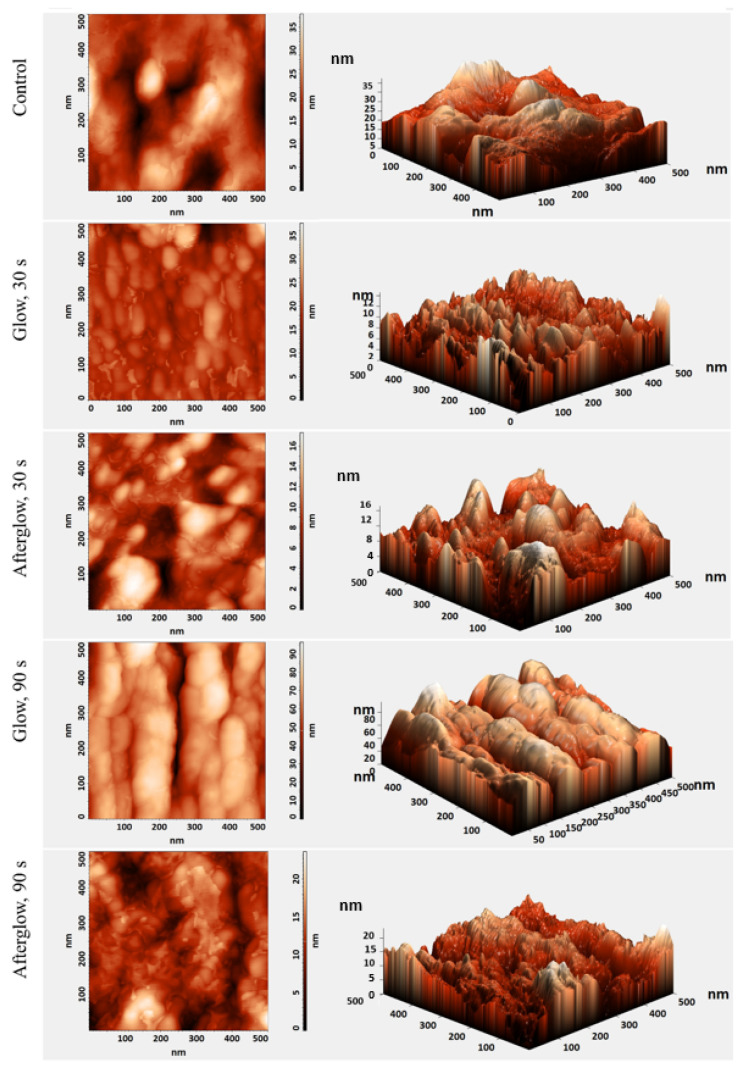
AFM images and 3D reconstruction of seed coat surface in untreated seeds and plasma-treated seeds in glow regime for 30 s and 90 s and afterglow plasma regime after 30 s and 90 s.

**Figure A2:**
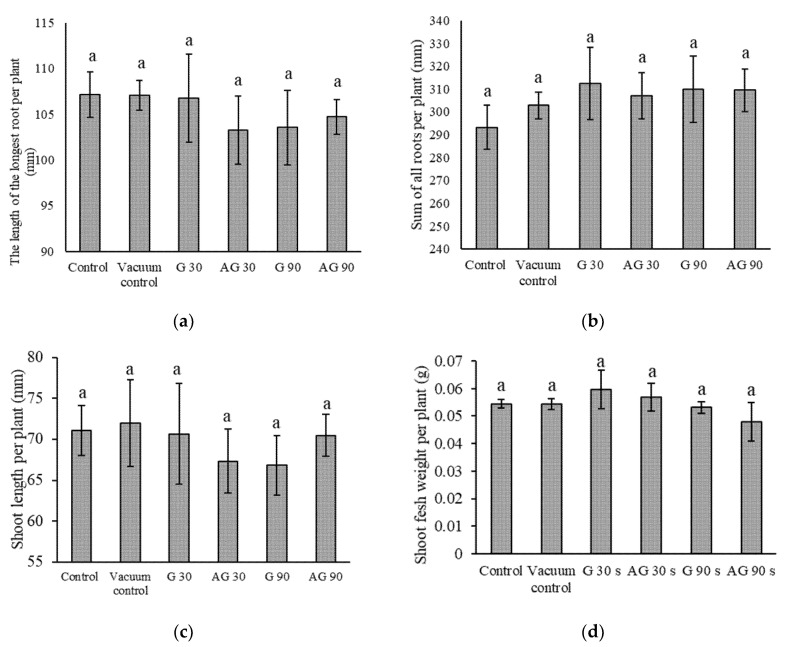
The average length of the longest root per plant (**a**), total sum of root length per plant (**b**), the average shoot length per plant (**c**), and the average fresh weight of the shoot (**d**). Seeds were treated in the glow plasma regime for 30 (G30) or 90 s (G90), and in the afterglow regime for 30 (AG30) or 90 s (AG90). Displayed values are the mean ± SE of three replications. Letter a indicate statistically significant differences among treatments according to Duncan’s test (*p* < 0.05).

## Data Availability

The data presented in this study are available on reasonable request from the corresponding author.

## Appendix A

In the Appendix A, Figure A1 represents all image data from AFM studies of the wheat surface and a 3D reconstruction of seed coat surface for all plasma treatments, as well as untreated seeds.

## Appendix B

Appendix of the results with no statistically significant differences of plasma-treated samples compared to control of the length of the longest root, the sum of root length, the average shoot length, and the average fresh weight of the shoot in Figure A2.
